# 2955. The Impact of the Healthcare Environment on the Nasal Microbiome in Children with Cancer

**DOI:** 10.1093/ofid/ofad500.194

**Published:** 2023-11-27

**Authors:** Jonathon C McNeil, Lauren M Sommer, Marritta Joseph, Jessica Runge, Marudhu Murugesan, Julienne Brackett, Amya Mitchell, Matthew Wilber, Ruth Ann Luna

**Affiliations:** Baylor College of Medicine, Houston, Texas; Baylor College of Medicine, Houston, Texas; Baylor College of Medicine, Houston, Texas; Baylor College of Medicine, Houston, Texas; Baylor College of Medicine, Houston, Texas; Baylor College of Medicine, Houston, Texas; Texas Children's Pediatrics, Houston, Texas; Texas Children's Pediatrics, Houston, Texas; Baylor College of Medicine, Houston, Texas

## Abstract

**Background:**

Children with cancer are at risk for healthcare associated infections (HAIs) given their immunosuppression as well as frequent hospitalizations and antibiotic and antiseptic exposure. The degree to which the healthcare environment impacts the flora of children with cancer and its subsequent role in infection is uncertain. We prospectively evaluated the nasal microbiome in children with malignancy relative to healthy children.

**Methods:**

Subjects were enrolled into two cohorts. The high risk cohort included children with a new diagnosis of malignancy recruited from the Texas Children’s Hospital Cancer Center. Children in the low risk cohort were otherwise healthy and enrolled from two Houston area general pediatrics clinics. Subjects had anterior nares swab samples obtained at 3-month intervals for one year. Genomic material was extracted from samples and the 16S rRNA V4 region was amplified and sequenced. Microbiome analysis was performed using QIIME 2, and comparisons in both bacterial diversity (alpha diversity via Shannon index) and composition (genus-level) were made based on cohort and time point. We present early data from the time of subject enrollment and first follow-up.

**Results:**

272 subjects were enrolled. Cohorts were similar in terms of demographics. No subjects were neutropenic at time of sample collection. High-risk subjects were significantly more likely to have received antibiotics (96.4% vs. 17.5%, p< 0.01) and/or chlorhexidine gluconate containing products (91.6% vs. 0%, p< 0.01) in the 90 days prior to enrollment. Within the high-risk cohort, 98.8% had received antineoplastic chemotherapy. Alpha diversity (Shannon index) was lower in the high risk cohort compared to the low risk cohort. In the high-risk cohort, the genera *Staphylococcus* and *Corynebacterium* were disproportionately represented relative to the low-risk cohort in which *Moraxella* predominated (**Figure 1**). Nasal microbiota were similar across cancer diagnoses.

**Figure:**
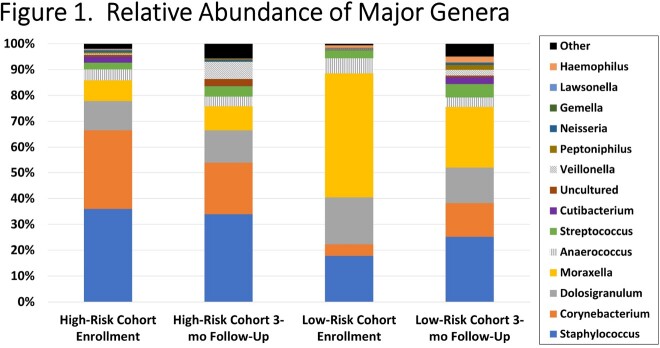

**Conclusion:**

There are significant changes in the nasal microbiota of children with cancer relative to healthy children. These findings suggest a high impact of healthcare exposure and specifically antibiotics and antiseptics on colonizing flora. Further work is needed to understand how these microbiome perturbations modify risk for HAI.

**Disclosures:**

**Jonathon C. McNeil, MD**, Allergan: Grant/Research Support|Nabriva Therapeutics: Grant/Research Support

